# Does Robotic Total Hip Arthroplasty Technology Have Any Benefit in Achieving Equal Limb Length via the Direct Anterior Approach?

**DOI:** 10.7759/cureus.92716

**Published:** 2025-09-19

**Authors:** Mawardi B Mustapha, Agir Muzaffer, Javad Parvizi, Ibrahim Tuncay

**Affiliations:** 1 Orthopedics, International Joint Center, Acibadem Maslak Hospital, Istanbul, TUR

**Keywords:** direct anterior approach, leg length discrepancy, orthopedic surgery, robotic-assisted surgery, total hip arthroplasty

## Abstract

Background

Leg length discrepancy (LLD) is a common and clinically significant complication following total hip arthroplasty (THA), potentially affecting functional outcomes and patient satisfaction. Robotic-assisted THA has emerged as a promising alternative to conventional techniques, offering enhanced precision in implant positioning. This study aims to evaluate whether robotic-assisted THA via the direct anterior approach provides significant advantages in achieving equal limb length compared to conventional techniques.

Methodology

A retrospective analysis was conducted among 200 patients with primary hip osteoarthritis who underwent THA via the direct anterior approach from February 2018 to August 2024. Of these, 100 patients underwent robotic-assisted THA, and 100 underwent conventional THA. Postoperative leg length measurements were obtained using standardized radiographs, and LLD was classified into mild (<5 mm), moderate (5-10 mm), and severe (>10 mm). Statistical analyses, including independent t-tests, chi-square tests, and analysis of covariance, were performed to compare LLD between the two groups while adjusting for age and body mass index (BMI).

Results

The robotic-assisted group demonstrated a significantly lower mean LLD compared to the conventional group (4.05 mm vs. 6.0 mm; p = 0.001). After adjusting for age and BMI, the difference remained significant (adjusted mean difference = -1.84 mm, 95% confidence interval = 0.638-3.03; p = 0.003). The rates of moderate-to-severe LLD were 55% in the conventional group and 44% in the robotic-assisted group, though this difference was not statistically significant (p = 0.23).

Conclusions

Robotic-assisted THA via the direct anterior approach significantly reduces the magnitude of LLD compared to conventional techniques while achieving similar efficacy in minimizing severe discrepancies. These findings highlight the potential benefits of robotic assistance in optimizing surgical precision, though further research is needed to justify its cost-effectiveness and impact on long-term functional outcomes.

## Introduction

Total hip arthroplasty (THA) is a well-established surgical intervention for patients suffering from debilitating hip joint disorders, primarily osteoarthritis [1,2]. With the aging population and rising prevalence of hip-related pathologies, the demand for THA has significantly increased [3]. While conventional techniques have historically dominated the field, advancements in surgical technology, particularly robotic-assisted surgery, have emerged as promising alternatives [4].

Conventional THA involves a manual approach where surgeons rely on their experience and anatomical landmarks to position the implants accurately. While most operations achieve satisfactory outcomes, achieving consistent leg length restoration remains a challenge. Variability in surgical technique and intraoperative factors can lead to unintended discrepancies in leg length, which underscores the need for improved precision in surgical procedures.

Leg length discrepancy (LLD) is a common complication following THA and can lead to a range of postoperative issues, including functional impairment, gait abnormalities, and decreased patient satisfaction [5]. LLD may result from various factors, including surgical technique, implant positioning, and the inherent anatomical variations among patients. Studies have reported that discrepancies greater than 1 cm can significantly affect a patient’s quality of life and may necessitate further interventions such as shoe lifts or revision surgery [6,7].

Robotic-assisted THA has been introduced as a potential solution to mitigate the risks associated with LLD [8]. Robotic systems provide enhanced visualization, precision, and reproducibility by allowing for preoperative planning and intraoperative adjustments based on real-time feedback. These systems can potentially reduce the variability associated with conventional techniques, leading to improved postoperative outcomes, including more accurate restoration of leg length. The direct anterior approach is a muscle-sparing technique commonly described as minimally invasive; it avoids detaching major muscles, resulting in faster rehabilitation and reduced postoperative pain [9]. However, the effectiveness of this approach in conjunction with robotic assistance compared to conventional methods remains inadequately explored.

Given the increasing adoption of robotic technologies in orthopedic surgery and the substantial impact that LLDs have on patient outcomes, it is critical to investigate whether robotic-assisted THA can provide superior results compared to conventional techniques when performed via the direct anterior approach. This study aims to evaluate whether robotic-assisted THA via the direct anterior approach provides significant advantages in achieving equal limb length compared to conventional techniques.

## Materials and methods

In this retrospective cohort study, the study population was patients with primary hip osteoarthritis. Consecutive cases that underwent THA via the direct anterior approach were reviewed from February 2018 to August 2024. All data were obtained from medical records. Institutional Review Board approval of the study was obtained from the Acibadem Mehmet Ali Aydinlar University Medical Research Ethics Committee (approval number: 2025-04/19).

The inclusion criteria were surgeries performed using either robotic-assisted or conventional techniques. Included patients needed to have postoperative pelvis radiographs to measure leg length. Exclusion criteria were patients with complex THA that required additional instruments or procedures (e.g., metal augment, bony procedure, spontaneous bilateral THA), patients with pre-existing conditions that affect leg length (e.g., congenital deformities, spinal conditions), and incomplete medical records or radiographic data. The study involved 200 patients, 100 in the robotic-assisted group and 100 in the conventional procedures group. From the medical records of the patients, demographic data such as age, body mass index (BMI), and sex were gathered.

**Figure 1:**
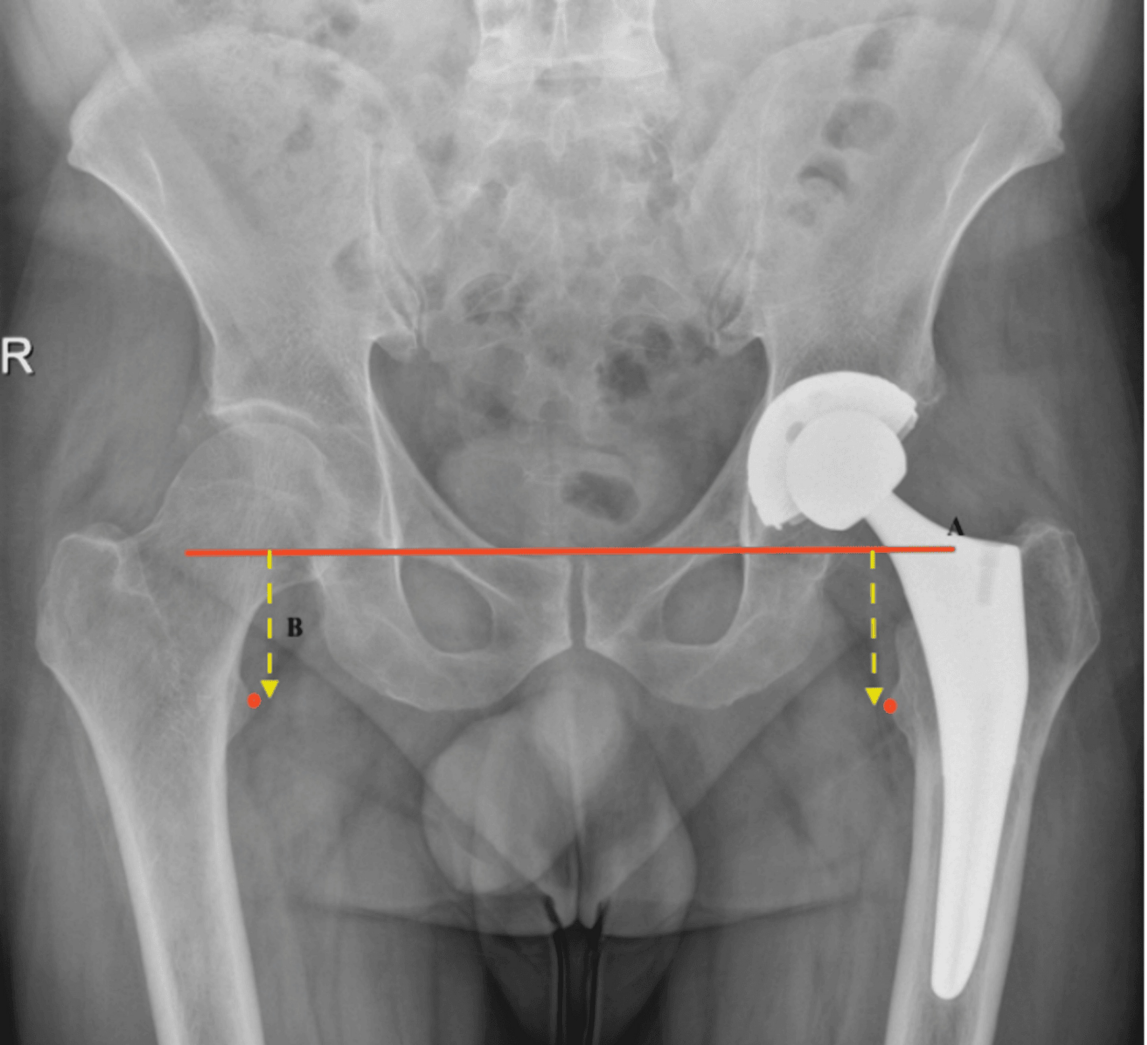
LLD measurement from AP view X-ray of the pelvis. (A) The inter-teardrop line. (B) The distance between the inter-teardrop line and the most prominent portion of the lesser trochanter. LLD = leg length discrepancy; AP = anteroposterior

Radiographic assessments were performed on standardized anteroposterior (AP) pelvic radiographs obtained two to three weeks postoperatively. LLD was evaluated using the trochanteric method, in which a horizontal inter-teardrop line is drawn on AP pelvis radiographs and the vertical distance to the most prominent point of the lesser trochanter is measured, as described by Germain et al. [10] (Figure 1).

There are various classifications of LLD based on severity and its effect in the literature [9]. The measurements in this study were categorized into the following three groups: minor LLD (less than 5 mm, generally considered within the normal range and may not cause significant symptoms), moderate LLD (between 5 and 10 mm, may cause functional symptoms such as limping or back pain), and severe LLD (greater than 10 mm, more likely to require intervention, such as a shoe lift or revision surgery).

The direct anterior approach was performed with the patient in the supine position on a standard operating room table, in accordance with the technique variations outlined by Connolly and Kamath [11]. Fluoroscopy was utilized for acetabular and femoral component placement.

Statistical analysis was performed using SPSS software version 22 (IBM Corp., Armonk, NY, USA). The LLD group was analyzed using the chi-square test and the independent t-test for age and BMI. Confounders of age and BMI were adjusted using analysis of covariance.

## Results

The overall mean age of the cohort was 62.6 years (range = 36-83 years). In subgroup analysis, the conventional group had a mean age of 60.89 years, while the robotic-assisted group had a mean age of 64.3 years. The mean BMI of all patients was 29.02 kg/m² (range = 17.7-57.7 kg/m²), with group-specific means of 29.37 kg/m² in the conventional group and 28.68 kg/m² in the robotic-assisted group. Of the 200 patients, 71 (35.5%) were male, and 129 (64.5%) were female. The conventional group comprised 32 men and 68 women, whereas the robotic-assisted group included 39 men and 61 women. These demographic characteristics are summarized in Table 1, with a statistically significant difference noted in mean age between the two groups.

**Table 1:** Patients’ characteristics between groups. ^a^: Independent t-test; ^b^: Chi-square test.

Characteristic	Conventional group	Robotic-assisted group	P-value	Test statistic
Age (mean, SD)	60.89 (10.25)	64.28 (10.15)	0.02^a^	-2.350
Body mass index (mean, SD)	29.37 (6.19)	28.68 (4.19)	0.36^a^	0.918
Gender (male/female)	32/68	39 / 61	0.3^b^	1.07

On comparing LLD without controlling other variables, there was a significant difference in LLD between conventional and robotic-assisted methods. A similar finding was reported after controlling for age and BMI. There was a significant difference in mean LLD, with the conventional group having a higher mean LLD than the robotic-assisted group (Tables 2, 3).

**Table 2:** Comparison of leg length discrepancy between conventional and robotic-assisted methods without adjustment of age and body mass index. ^a^: Independent t-test

Method	n	Mean (SD)	Mean difference (95% CI)	P-value	Test statistic
Conventional	100	6.0 (4.92)	1.95 (0.79–3.11)	0.001^a^	3.308
Robotic-assisted	100	4.05 (3.24)			

**Table 3:** Comparison of leg length discrepancy between conventional and robotic-assisted methods with adjustment of age and body mass index. ^a^: Adjusted mean difference (95% confidence interval) with Bonferroni adjustment; ^b^: analysis of covariance (adjusted for age and body mass index).

Method	n	Mean	Mean difference (95% CI)	P-value	Test statistic
Conventional	100	5.94 (5.10–6.77)	-1.84 (0.638–3.03)^a^	0.003^b^	3.466
Robotic-assisted	100	4.10 (3.27–4.94)			

The rate of moderate-to-severe LLD was 55% in the conventional group and 44% in the robotic-assisted group. Although the incidence was 11% higher, the difference was not statistically significant (Table 4).

**Table 4:** Severity of leg length discrepancy (LLD) between methods. ^a^: Chi-square test.

Severity of LLD	Conventional group (n = 100)	Robotic-assisted group (n = 100)	P-value	Test statistic
Mild	45%	56%	0.23^a^	2.931
Moderate	26%	24%		
Severe	29%	20%		

## Discussion

Although THA is widely regarded as one of the most successful surgical procedures, LLD remains a common complication, particularly with conventional techniques [5]. Accurate restoration of leg length requires precise acetabular anteversion and inclination; however, due to pelvic mobility, complex anatomical variations, and variability in surgical expertise, free-hand component positioning is prone to errors [12,13].

THA can be performed using several approaches; however, the posterior, direct lateral, and direct anterior approaches remain the most commonly utilized worldwide [14]. The anterior approach, as applied in this study, is typically performed with the patient in a supine position and allows the use of intraoperative fluoroscopy. In contrast, the anterolateral and posterior approaches are generally performed in the lateral decubitus position, where fluoroscopic assistance is less frequently utilized [15]. Over the past three decades, robotic systems have been introduced into THA with the aim of reducing human error in implant positioning and thereby improving surgical precision and patient outcomes.

In the present cohort, robotic-assisted THA was associated with a smaller mean LLD compared to the conventional direct anterior approach, suggesting that robotic technology contributes to improved accuracy in restoring limb length. However, both techniques were similarly effective in reducing the occurrence of moderate-to-severe discrepancies. Although patients in the robotic-assisted group were significantly older, adjustment for age confirmed that the observed differences in LLD remained significant. These findings are consistent with previously published research comparing robotic and conventional THA.

For instance, Ma et al., in a propensity score-matched analysis, reported improved leg length restoration with robotic-assisted THA, whether performed through a posterior or anterior approach, compared to manual techniques in patients with advanced femoral head osteonecrosis [16]. Similarly, another study evaluating robot-assisted posterior, fluoroscopy-guided anterior, and conventional posterior approaches demonstrated that while all were effective in minimizing LLD, the robot-assisted posterior approach achieved the lowest mean discrepancy, supporting our observations [8].

A meta-analysis by Kumar et al. further reinforced these findings, showing a significant reduction in LLD with robotic-assisted THA across nine studies. Their analysis also demonstrated that robotic systems increased the proportion of acetabular cups placed within Lewinnek’s and Callanan’s safe zones. However, this came at the expense of longer operative times. Importantly, they found no significant differences in perioperative complication rates, revision surgery, or long-term functional outcomes between robotic-assisted and conventional techniques [17]. In line with these findings, a systematic overview of meta-analyses by Kort et al. concluded that robotic-assisted THA improves implant positioning and LLD correction but is associated with increased operative duration and a higher risk of heterotopic ossification, dislocation, and revision [4].

This study has several strengths, including a relatively large sample size derived from two centers and the use of blinded assessors for radiographic measurements. Nonetheless, some limitations should be acknowledged. First, as a retrospective analysis, randomization was not possible, and the choice between robotic-assisted and conventional THA was influenced by surgeon preference and institutional practices, raising the possibility of selection bias. Second, although standardized AP pelvic radiographs were used, variations in patient positioning, such as pelvic tilt or leg rotation, may have influenced the accuracy of LLD measurements, and differences in imaging technique across centers could have introduced additional variability. Lastly, the study did not evaluate functional outcomes or patient-reported measures. As some individuals with minimal discrepancies may still experience symptoms, while others with larger discrepancies may remain asymptomatic, the absence of functional data limits the ability to assess the true clinical relevance of LLD.

## Conclusions

This retrospective study demonstrates that robotic-assisted THA performed via the direct anterior approach achieves significantly lower mean LLD compared with the conventional technique, even after adjusting for potential confounders such as age and BMI. While both methods were similarly effective in reducing the incidence of moderate-to-severe discrepancies, robotic assistance provided superior precision in minimizing overall limb length inequality. These findings support the role of robotic technology in enhancing surgical accuracy during THA. However, given the higher costs and increased operative time associated with robotic systems, further prospective, randomized studies with long-term follow-up are needed to determine their cost-effectiveness, functional benefits, and overall impact on patient outcomes.

## References

[1] Learmonth ID, Young C, Rorabeck C (2007). The operation of the century: total hip replacement. Lancet.

[2] Migliorini F, Cuozzo F, Oliva F, Eschweiler J, Hildebrand F, Maffulli N (2023). CT-based navigation for total hip arthroplasty: a meta-analysis. Eur J Med Res.

[3] Pabinger C, Lothaller H, Portner N, Geissler A (2018). Projections of hip arthroplasty in OECD countries up to 2050. Hip Int.

[4] Kort N, Stirling P, Pilot P, Müller JH (2021). Clinical and surgical outcomes of robot-assisted versus conventional total hip arthroplasty: a systematic overview of meta-analyses. EFORT Open Rev.

[5] Desai AS, Dramis A, Board TN (2013). Leg length discrepancy after total hip arthroplasty: a review of literature. Curr Rev Musculoskelet Med.

[6] Edeen J, Sharkey PF, Alexander AH (1995). Clinical significance of leg-length inequality after total hip arthroplasty. Am J Orthop (Belle Mead NJ).

[7] Konyves A, Bannister GC (2005). The importance of leg length discrepancy after total hip arthroplasty. J Bone Joint Surg Br.

[8] El Bitar YF, Stone JC, Jackson TJ, Lindner D, Stake CE, Domb BG (2015). Leg-length discrepancy after total hip arthroplasty: comparison of robot-assisted posterior, fluoroscopy-guided anterior, and conventional posterior approaches. Am J Orthop (Belle Mead NJ).

[9] Post ZD, Orozco F, Diaz-Ledezma C, Hozack WJ, Ong A (2014). Direct anterior approach for total hip arthroplasty: indications, technique, and results. J Am Acad Orthop Surg.

[10] Germain E, Lombard C, Boubaker F (2022). Imaging in hip arthroplasty management—part 1: templating: past, present and future. J Clin Med.

[11] Connolly KP, Kamath AF (2016). Direct anterior total hip arthroplasty: literature review of variations in surgical technique. World J Orthop.

[12] Gonzalez Della Valle A, Shanaghan K, Benson JR, Carroll K, Cross M, McLawhorn A, Sculco PK (2019). Pelvic pitch and roll during total hip arthroplasty performed through a posterolateral approach. A potential source of error in free-hand cup positioning. Int Orthop.

[13] Murray DW (1993). The definition and measurement of acetabular orientation. J Bone Joint Surg Br.

[14] Angerame MR, Holst DC, Yang CC (2018). Surgical approaches for total hip arthroplasty. Ann Joint.

[15] Kennon RE, Keggi JM, Wetmore RS, Zatorski LE, Huo MH, Keggi KJ (2003). Total hip arthroplasty through a minimally invasive anterior surgical approach. J Bone Joint Surg Am.

[16] Ma M, Song P, Zhang S, Kong X, Chai W (2023). Does robot-assisted surgery reduce leg length discrepancy in total hip replacement? Robot-assisted posterior approach versus direct anterior approach and manual posterior approach: a propensity score-matching study. J Orthop Surg Res.

[17] Kumar V, Patel S, Baburaj V, Rajnish RK, Aggarwal S (2023). Does robotic-assisted surgery improve outcomes of total hip arthroplasty compared to manual technique? A systematic review and meta-analysis. Postgrad Med J.

